# Estimation of direct economic and productive losses due to abortions caused by *Neospora caninum* in the primary dairy sector of Uruguay

**DOI:** 10.3389/fvets.2025.1502742

**Published:** 2025-03-26

**Authors:** Miguel Carrillo Parraguez, Eduardo Ponssa, Darío Caffarena, Jorge Artagaveytia, Fernando Sotelo, Santiago Fariña, Alejandro Mendoza, Federico Giannitti

**Affiliations:** ^1^Plataforma de Investigación en Salud Animal, Instituto Nacional de Investigación Agropecuaria (INIA), Estación Experimental La Estanzuela, Colonia, Uruguay; ^2^Escuela de Medicina Veterinaria, Facultad de Ciencias de la Vida, Universidad Andrés Bello, Concepción, Chile; ^3^Facultad de Ciencias Veterinarias, Universidad Nacional del Centro de la Provincia de Buenos Aires (UNCPBA), Tandil, Argentina; ^4^Unidad Académica Salud de los Rumiantes, Departamento de Producción Animal, Facultad de Veterinaria, Universidad de la República, Montevideo, Uruguay; ^5^Instituto Nacional de la Leche (INALE), Montevideo, Uruguay; ^6^Instituto Nacional para el Mejoramiento Lechero (MU), Montevideo, Uruguay; ^7^Sistema Lechero, Instituto Nacional de Investigación Agropecuaria (INIA), Estación Experimental La Estanzuela, Colonia, Uruguay

**Keywords:** abortion, bovine neosporosis, dairy production, economic losses, lost profits, Uruguay

## Abstract

Despite its sociocultural and economic importance, dairy farming in Uruguay has not experienced a significant increase in cattle stocks in recent decades, partly due to low reproductive efficiency and a high risk of calf mortality. *Neospora caninum* is one of the primary abortive pathogens in dairy cattle; however, the economic losses caused by neosporosis in Uruguay remain uncalculated. This study aimed to assess the direct annual economic losses for the primary dairy sector (dairy farmers) due to abortions resulting from bovine neosporosis in the Uruguayan herd. An adjusted bioeconomic model was used to estimate the economic losses from early (second gestational trimester) or late (third gestational trimester) abortions, considering the Present Value (PV) of future production loss due to these abortions after deducting production costs (lost profits). The average economic loss per abortion due to neosporosis was US$ 868 (range 605–1,162) if the aborting cow remained in the herd without any additional abortions throughout its productive life and US$ 1,866 (range 782–2,825) if the cow was culled after the abortion. Individual losses varied based on the gestational stage and the age of the aborting cow, with the worst-case scenario being a late abortion in first-pregnancy heifers. The annual number of abortions due to neosporosis was estimated by considering the stock of cows and heifers of reproductive age at risk of abortion (pregnant) and the national seroprevalence of *N. caninum* in dairy herds, accounting for 90% vertical transmission and 10% horizontal transmission, with aborting seropositive dams representing 15 and 35%, respectively. The economic losses for the national herd were calculated by multiplying individual losses by the number of abortions across various simulated scenarios (second or third gestational trimester), adjusted according to sub-scenarios (with and without culling of the aborted cow). The estimated economic losses for the primary sector due to abortions occurring in 2018 totaled nearly US$ 12 million, which is attributed to the loss of offspring, delayed lactation, reduced productive life (fewer days in milk production/days of life), and early culling. These estimated losses reflect only the primary sector, excluding the secondary (industrial) and tertiary (services) sectors. In physical terms, abortions associated with neosporosis caused a total loss of 62 million L of milk, which is 3.3% of the volume industrialized annually in the country. The estimated losses did not account for profits lost by dairy farmers from beef production (i.e., breeding, fattening, and sale for slaughter of male calves from dairy breeds lost due to neosporosis) nor any indirect losses (for example, the loss of genetic merit, stunted growth of the national dairy stock, etc.). The characteristics of Uruguay’s grazing dairy production system require a tailored approach to estimating the economic impact of bovine diseases. This information can be used by farmers, veterinarians, and policymakers to evaluate the cost–benefit of implementing control and prevention strategies for bovine neosporosis at both the farm and national levels.

## 1 Introduction

Dairy production is socioeconomically significant in Uruguay. From 2010 to 2020, the volume of industrialized milk increased by approximately 34% (1). However, during this same period, there was a 1.9% decline in the total number of dairy cattle, including a specific decrease of 2.3% in the adult dairy cow population (1). This reduction in the dairy herd can be partly attributed to poor reproductive performance (2–4), which is crucial for the profitability of dairy farming.

Reproductive diseases in livestock lead to significant economic losses for the dairy industry (5). In Uruguay, there are no official data on the reproductive efficiency of the dairy herd at the national level; however, an evaluation of 26 commercial dairy farms in 2018 indicated that approximately 15% of cows experienced gestational losses (Gustavo Gastal, INIA La Estanzuela, personal communication, 2022). *Neospora caninum* has been identified as a major cause of abortions in cattle in Uruguay (6–9) and has a high prevalence at both the animal and herd levels (10). From an economic perspective, a disease is considered a disturbance in the productive system that results in the additional use of resources or reduced product generation (11). The economic and productive effects of neosporosis include abortion, decreased milk production (fewer days in milk during the productive life), increased risk of culling, reduced birth rates, and the limited availability of replacement heifers (5, 12).

The economic losses due to bovine neosporosis have been estimated to be in the billions of United States dollars (US$) globally, ranging from tens to hundreds of millions of US$ in South American countries such as Argentina and Brazil (13, 14). Models simulating the epidemiological dynamics of neosporosis (15), quantifying the economic losses (13, 15), and assessing the effects of control strategies along with their associated costs (16, 17) have been developed and implemented. This study aimed to estimate the economic and productive losses in the primary dairy sector due to abortions associated with neosporosis that occurred in 2018 in Uruguay, employing a methodological approach that was not previously applied to this disease.

## 2 Materials and methods

A deterministic, dynamic individual bioeconomic model that stimulates the productive life cycle of a dairy cow from birth to culling was developed in Excel^®^ and calibrated using data from the average dairy cow at the “*Instituto Nacional para el Mejoramiento Lechero*” (MU) as well as information on the national dairy system provided by the Uruguayan “*Instituto Nacional de la Leche*” (INALE) and the “*Instituto Nacional de Investigación Agropecuaria*” (INIA). The model is primarily based on the assumption that the economic impact of abortions on dairy systems results in an anticipated future production loss (lost profits).

### 2.1 Bioeconomic model overview

The model was based on the following stages of the production cycle: Calf Rearing (CR), three phases of Replacement Heifer Rearing (RHR 1–3), Lactation 1, Dry Period (DP) 1, and Lactation “n”, and DP “n.” These stages were delineated by the following events: birth (beginning of CR), weaning (beginning of RHR 1), puberty (beginning of RHR 2), pregnancy (beginning of RHR 3), parturition 1 to “n” (beginning of Lactation 1 to “n”), and drying off (start of DP 1 to “n”). At each stage, differences in diet, labor, health management, weight gain, and milk production were established according to the stage of lactation. A gestation length of 283 days was considered (18). The age at first conception was estimated as the difference between the length of gestation and the age at first calving, using average values from dairy cattle in Uruguay (Fernando Sotelo, MU, personal communication, 2019). The proportion of calves born male or female was assumed to be 50%, allowing for an average value based on the sale of both male and female calves. We assumed that body weight increased from birth until the DP 1 stage, after which it stabilized at the adult weight. Income from milk production was estimated based on yield per lactation, using local average production values multiplied by the price of milk paid to farmers in the local market (0.30 US$/L) (19).

The impact of abortion was evaluated by accounting for the differences between the base scenario (no abortion) and different abortion scenarios, considering whether the abortion occurred early (in the second gestational trimester) or (late in the third gestational trimester), across different pregnancies (first, second, third, and so on). Consequently, the economic and productive differences between the base and simulated scenarios stem from the simulated abortion event and the disruptions it causes in productive life. These disruptions are reflected in the start, end, and duration of each life stage (temporal variables), as well as in the beginning, end, and duration of the reproductive stages of pregnancy and open days (reproductive variables).

To assess the economic impact of abortion, various response variables were used, including Accumulated Balance, Present Value (PV), Internal Rate of Return (IRR), and Payback Period. Detailed definitions of these variables are presented in Supplementary material I. The difference in PV between the base scenario and each abortion scenario reflects, in a broad sense, the economic impact of abortion. The economic loss due to abortion was determined by calculating the PV of future lost milk production minus the direct costs avoided as a result of this event (lost profits). Therefore, the economic losses from abortion were estimated by calculating the difference between the PV of a cow in the base scenario (no abortion) and the abortion scenario (20). The individual economic loss resulting from the abortion, along with the culling of the aborted cow, was estimated as the income from the sale of the cow for slaughter minus its loss of value, based on the method proposed by Chi et al. (21). In our study, the value of the culled cow was determined from the PV of a cow in the base scenario at the age specific to each abortion scenario, while the income from sales for slaughter was calculated based on an average of US$ 600 per cow sold, according to Caffarena (18).

Productive losses at the individual level were assessed based on the loss of offspring and the decline in lifetime milk production relative to the base scenario. At the national level, productive losses accounted for the annual number of abortions due to neosporosis, utilizing a modified formula from Moore et al. (13) and Reichel et al. (14).

The model considers the 7-year (2,544-day) lifespan of an average dairy cow in Uruguay (MU) and projects the losses due to abortion in a PV for each scenario once this lifespan ends. Consequently, the losses from abortions that occurred in 2018 will accumulate until 2025.

### 2.2 Input data

#### 2.2.1 Generic input data

The generic input data for the base and abortion scenarios, as well as the input data for the productive stages (which include, for example, data on feeding management for each stage of the animal’s life) for the base scenario, are detailed in Supplementary material II. Other input variables, including historical prices and investments in facilities and equipment, were obtained from INALE, MU, and INIA and are the same as those used by Caffarena (18).

#### 2.2.2 Reproductive input data

The age at first pregnancy was 724 days (24.1 months), while the age at first calving was 1,006 days (33.5 months). The birth-conception intervals (open days) were 175, 168, 166, and 180 days for the first, second, third, and fourth calvings, respectively. Based on these values, the calving intervals were 458, 451, and 449 days for the first-to-second, second-to-third, and third-to-fourth calvings, respectively. Since the age of culling remained constant at 2,544 days (reflecting the average culling age of dairy cows in Uruguay -MU-), the interval between the fourth parturition and culling was 180 days.

The simulated abortion scenarios were based on the assumption that a single abortion could occur at any point during the animal’s lifespan, which could potentially happen in different pregnancies (first, second, third, or fourth) and at various stages of gestation. These stages were identified as early abortion (135 days of gestation, second trimester) and late abortion (225 days of gestation, third trimester), which represent the gestational window in which most abortions caused by *N. caninum* occur (12, 22–24). The open days following the abortion in each scenario aligned with those of the corresponding lactations in the base scenario, as the abortion interrupted the pregnancy and initiated the open period without leading to lactation or the birth of a calf. Consequently, the abortion event affected the calving interval for lactations in the second, third, and fourth pregnancy scenarios while extending the age at first calving in the first pregnancy scenarios.

In the simulated abortion scenarios, the abortion-conception interval matched the birth-conception interval that would have occurred in a successful pregnancy. However, the abortion scenarios featured an early onset of the birth-abortion-to-conception interval, which also lasted the same duration as mentioned previously for each respective pregnancy.

#### 2.2.3 Input data by production stages

The input data for milk production was maintained in terms of both duration and production volume. The first lactation lasted 395 days, with a daily production of 17.43 L, while the second lactation lasted 389 days, with a daily production of 19.98 L. The third lactation was incomplete, lasting 329 days in the early abortion scenario and 239 days in the late abortion scenario, with a daily production of 20.86 L. In the model, lactations were divided into four phases: the first three lasting 90 days each, followed by a final phase of 125 or 119 days. In the abortion scenarios, the fourth lactation was absent (as one lactation was lost), lasting 59 or 90 days. The equations and additional input data regarding the duration of different lactation stages in each scenario are provided in Supplementary material II.

### 2.3 Number of cows at risk, abortions, culling, and distribution of scenarios and sub-scenarios

The estimate of the number of abortions was based on a modification of the formula used by Moore et al. (13) and Reichel et al. (14), which accounts for differences in the proportions of cattle that abort after vertical or horizontal transmission of *N. caninum*, as proposed by Thurmond and Hietala (25) and McAllister et al. (26), respectively.


$$NcA\left(n\right)=\left(n\times PP\right)\times SP\times \mathrm{NcPA}$$

*NcA(n)*: Number of abortions due to *N. caninum.*

*n:* Cows and heifers in reproductive age.

*PP:* Pregnancy proportion (proportion of cows and heifers of reproductive age that become pregnant annually).

(*n* × *PP*): Number of cows and heifers at risk of abortion (pregnant).

*SP:* Seroprevalence.

*NcPA:* Proportion of cattle that abort after vertical or horizontal transmission of *N. caninum.*

In 2018, the number of female dairy cattle of reproductive age was 430,000, comprising 310,000 cows and 120,000 heifers (27). The annual pregnancy rate was estimated at 85 and 75% for heifers and cows, respectively (Gustavo Gastal, INIA La Estanzuela, 2022, personal communication). Considering a nationwide seroprevalence of *N. caninum* of 22% at the animal level (10), we estimated that 73,590 pregnant cows and heifers were seropositive in 2018. Of these, we assumed that 90% (66,231) were infected through vertical (congenital) transmission, while the remaining 10% (7,359) were infected horizontally. We also assumed that the proportion of seropositive animals that aborted was 15% for congenitally infected dams (25) and 35% for those that acquired the infection horizontally (26). Based on these estimates, vertical and horizontal transmission resulted in 9,935 and 2,576 abortions, respectively, totaling 12,510 abortions due to neosporosis in 2018.

The distribution of abortion scenarios due to neosporosis was established in equal proportions of 25% for heifers (first pregnancy) and cows in their second, third, and fourth pregnancies. Additionally, 50% of the cases in each of these categories were classified as early (135 days) or late (225 days) gestation abortions. This uniform distribution across different parities and gestation periods was determined based on the current heterogeneity of results in studies examining the attributable risk of abortion in cows seropositive for *N. caninum* (25, 28, 29) and the lack of specific data in Uruguay, where the disease is endemic and transmitted both vertically and horizontally.

To estimate the economic losses due to neosporosis resulting from the early culling of aborted cows, the different abortion scenarios were divided into two mutually exclusive sub-scenarios: one where the cow or heifer remained in the herd until the end of its lifespan after the abortion and another where it was immediately culled and sold for slaughter. These outcomes were based on a study by Thurmond and Hietala (30), which indicated that 8% of the seropositive cows that aborted were culled, while the remaining 92% were retained in the herd. Therefore, in our study, out of a total of 12,510 cows that were aborted across various scenarios, 11,509 were kept in the herd until the end of their productive life and 1,001 were immediately culled for slaughter.

## 3 Results and discussion

Although several studies have estimated economic losses due to neosporosis in cattle, most have been conducted by considering total losses minus the profit from selling the aborted cow, expressed in constant dollar values (13–15, 17, 21, 31). In contrast, this study estimates economic loss by calculating the differences between scenarios for response variables such as Present Value, Internal Rate of Return, and Payback Period, based on a model that first determines cash flow for each stage of productive life. This approach is novel in the context of neosporosis, allowing for more precise estimates by considering time effects, discounting production, management, and investment costs, and facilitating the construction of sub-scenarios for culling or maintaining the cow in the herd after the abortion.

### 3.1 Economic response variables and productive variables for all scenarios

**Figure 1:**
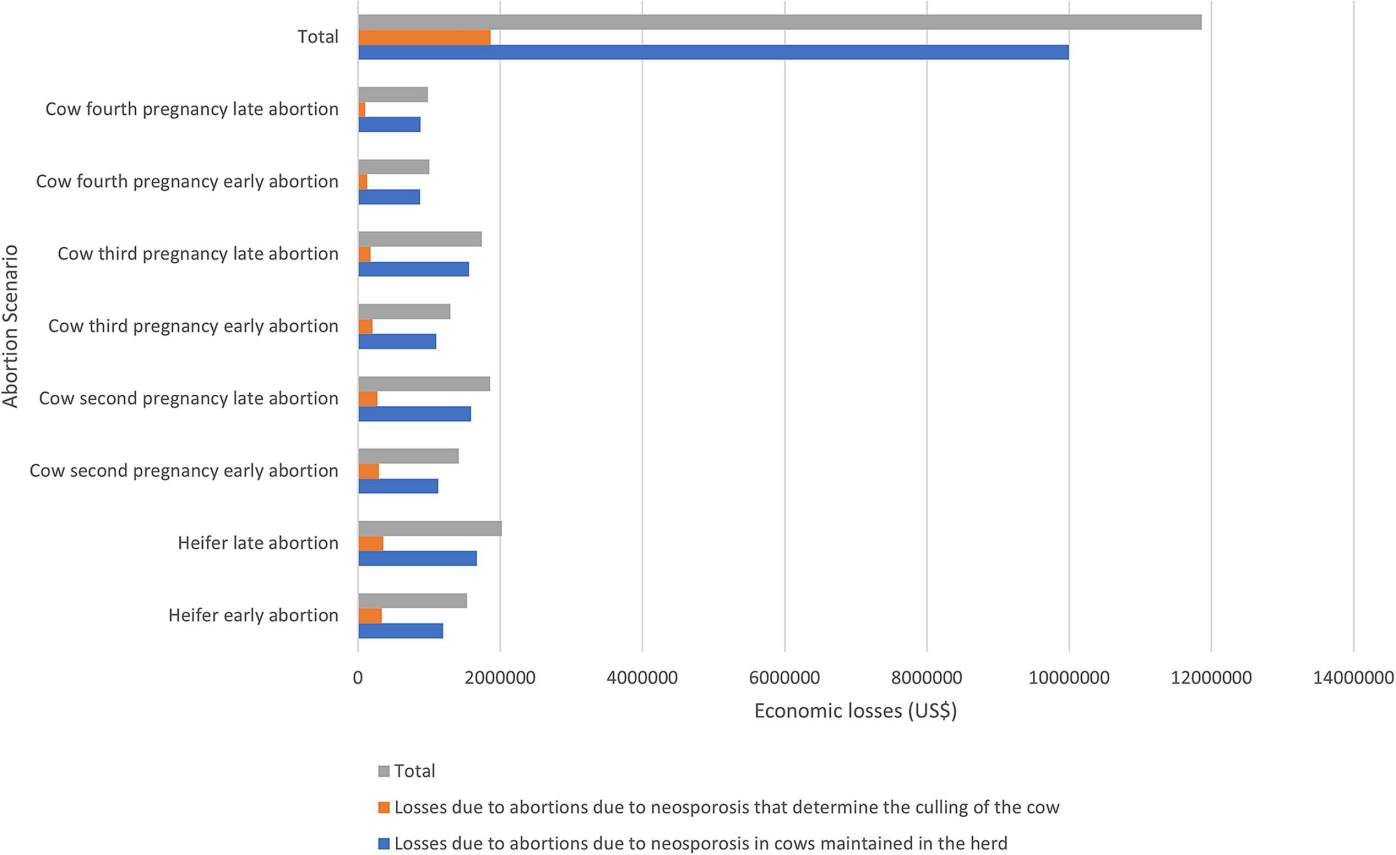
PV based on the scenario in US$ per cow. Historical trends in the PV of the base scenario (no abortion), and various scenarios involving abortions are shown. The abortion scenarios display trends similar to the base scenario regarding trajectory; however, they generally do not reach the same maximum values for each productive stage as the base scenario. In some scenarios, the maximum PV is delayed by several months. The fourth pregnancy scenarios (gray and light blue) overlap. In the abortion scenarios, we assumed that a single abortion occurs during the animal’s lifetime, with the aborted heifer or cow remaining in the herd until the end of its productive life.

In the base scenario, the PV peaked at US$ 3,629 at 34 months of age (Figure 1) after all rearing costs had been incurred and just before income from dairy production was generated. Following this peak, the PV declined but showed a slight increase at the beginning of each new lactation. This trend can be attributed to declining expenses from breeding and rearing as income from dairy production approaches. As each lactation progresses, the PV declines due to the decreasing future milk production over the remaining productive life. However, at the beginning of each new lactation, the PV of the future cash flow temporarily rises due to the expected increase in production to boost (time effect) and the additional income of US$ 61 from the sale of the calf born before continuing its downward trend.

In general, for this response variable, the abortion scenarios followed similar trends to the base scenario regarding trajectory, though on a smaller scale, not achieving the maximum equivalent values for each productive stage compared to the base scenario. In the “early” and “late” abortion scenarios in heifers, the PV peaked at 44 and 47 months, with values of US$ 3,169 and US$ 2,942, respectively. Conversely, in the second, third, and fourth pregnancy abortion scenarios, the maximum values were achieved at month 34, but they were at least US$ 500 below the maximum PV of the base scenario, with the fourth pregnancy abortion scenario being the closest to it (Figure 1). This is because the fourth pregnancy abortion scenarios most closely resembled the base scenario in terms of productive life history, allowing for the potential to compensate over time for all historical expenses in the cow’s life.

The base scenario had an IRR of 42.7%, while all abortion scenarios exhibited a lower IRR, indicating that they were less profitable, which aligns with the PV differences described above. For the early and late abortion scenarios, the IRR was 31 and 27% in heifers, 36 and 33% in second-pregnancy cows, 39 and 37% in third-pregnancy cows, and 41% for both in fourth-pregnancy cows. The least profitable abortion scenarios occurred in heifers, while those in fourth-pregnancy cows were closest to the base scenario (assuming only one abortion over a lifetime and that the aborted cow remained in the herd until the end of its productive life). These findings reaffirm the negative effect of a single abortion on the economic and productive performance of the cow.

The Payback Period occurred at 42.9 months of age in the base scenario and across all abortion scenarios, except for heifers, where it took place at 56.9 and 62.5 months of life for early and late abortions, respectively. This is because, in the base scenario and all abortion scenarios following the first lactation, the payback resulted from income generated by dairy production, and was completed at nearly half of the first lactation, offsetting the historical expenses of prior stages.

In the heifer abortion scenarios, since the abortion did not result in offspring or initiate the first lactation, production income was delayed, and consequently, the Payback Period was extended along with the accumulation of maintenance costs for the unproductive heifer. In contrast, in the abortion scenarios, the Payback Period was not delayed, as both the first calving and the first lactation were successful. However, subsequent lactations were affected, impacting other economic variables.

To the best of our knowledge, there are no studies assessing the effect of abortions caused by *N. caninum* on the economic variables we evaluated in this study (PV, IRR, and Payback Period).

The productive response variables included lifetime milk production and the number of offspring. In the base scenario, four calves and four lactations were produced; however, the last lactation lasted only 180 days, as the age at culling was kept constant to reflect the average lifespan of a dairy cow in Uruguay. Each abortion scenario resulted in the loss of one calf and lactation. In the heifer scenarios, abortion occurred during the first pregnancy, leading to the loss of the first calf and a delay in the onset of the first lactation.

In abortion scenarios during the second pregnancy in cows, abortions occurred in the first lactation. While the first lactation remained unaffected, the second calf was lost, and the onset of the second lactation was delayed.

**Figure 2:**
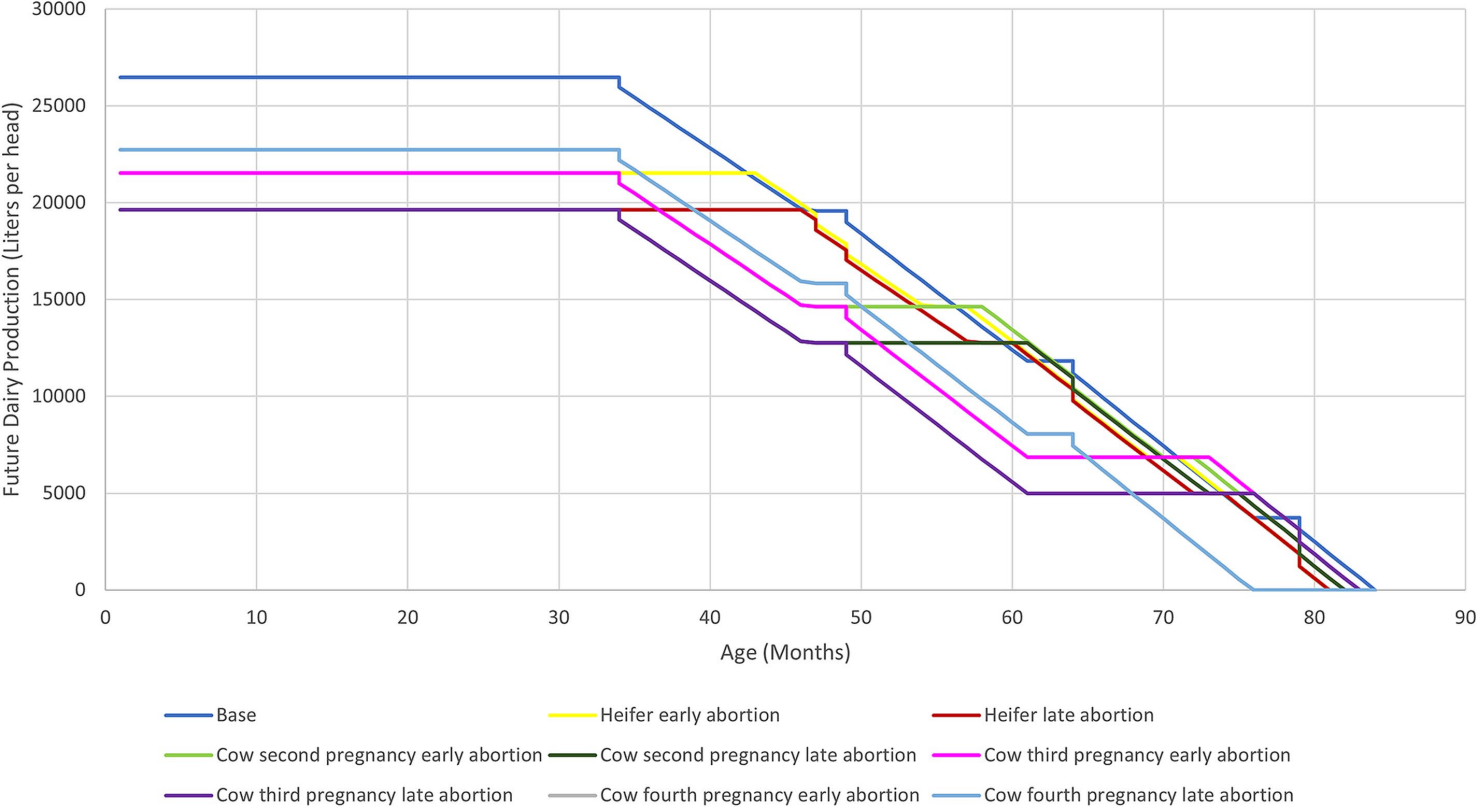
Dairy production in liters per cow based on the scenario. Historical trends of milk production under the base scenario (no abortion) and various abortion scenarios are presented. The abortion scenarios reveal trends similar to the base scenario in trajectory, but they generally did not reach the same maximum values for each productive stage when compared to the base scenario. The closest values were observed in the fourth pregnancy abortion scenarios, while the most distant values were found in the first pregnancy scenarios (heifers). The fourth pregnancy scenarios (gray and light blue) overlap. In the abortion scenarios, we assumed a single abortion during the animal’s lifetime, with the aborting heifer or cow retained in the herd until the end of its productive life.

In abortion scenarios during the third pregnancy, abortions took place in the second lactation. As a result, the first and second lactations were unaffected, but the third calf was lost, and the onset of the third lactation was delayed. Finally, in abortion scenarios during the fourth pregnancy, abortions occurred in the third lactation. The first, second, and third lactations were unaffected, but the fourth calf and lactation were lost. However, this productive loss was lower compared to the others. Trends in lifetime milk production in the base and abortion scenarios are shown in Figure 2.

### 3.2 Differences at each stage in the results of various scenarios and sub-scenarios of abortion due to neosporosis at the individual level for temporal, productive, reproductive, and economic variables

#### 3.2.1 Calf Rearing, Replacement Heifer Rearing 1, 2, and 3 with the first pregnancy

The simulated abortion across various scenarios had several effects on temporal, productive, reproductive, and economic variables. Regarding the CR and RHR stages 1 and 2, no differences were observed in the abortion scenarios compared to the base scenario or among abortion scenarios, as there were no pregnancies in these stages. In stage RHR 3, differences were evident in the “early” and “late” abortion scenarios for heifers compared to the base scenario. In these two scenarios, the first pregnancy was interrupted on days 135 or 225 of gestation, respectively (days 858 and 948 of life), which delayed the onset of the first lactation, extending the duration of stage RHR 3 (normally 276 days) to 585 and 675 days of life. Thus, the RHR 3 stage was extended by 309 and 399 days for each abortion scenario in the first pregnancy.

In the first pregnancy scenarios involving abortion, the average calving interval for early and late abortions was 409 and 379 days, respectively. These intervals differed by −42 and −72 days compared to the base scenario, which had an average calving interval of 451 days. In other scenarios, the average calving interval increased to 512 days for both early and late abortions, showing a difference of 61 days from the base scenario and indicating an overall increase in this indicator throughout the lifespan compared to the base scenario.

The estimated individual economic losses for early and late abortions in the first pregnancy were US$ 832 and US$ 1,162, respectively (Table 1). The differences between these two scenarios are due to the 3-month period that separates early from late abortions, representing an additional US$ 330.51 in the late abortion scenario. These estimates of individual losses assume that the animal remains in the herd (no culling), that the next successful pregnancy occurs under the same conditions as in the base scenario, and that no other abortions occur during its lifetime. If the aborted cow is immediately culled, the economic losses for these individual scenarios of early and late abortion amount to US$ 2,702 and US$ 2,825, respectively (Table 1).

**Table 1:** Extension of productive stages and results of individual economic and productive losses based on simulated scenarios and sub-scenarios.

Scenario		Stage and days of life (d) in which abortion occurs	Days of extension (d) of the stage due to abortion compared to the base scenario	Economic loss maintaining the cow in the herd (US$)	Economic loss culling the cow (US$)	Lifetime milk production (L) according to scenario	Lifetime milk production (L) lost due to abortion***
Base scenario (no abortion)		NA	0	0	0	26,474	0
Heifer (first pregnancy)	Early abortion*	RHR3, 858 d	309 d in RHR3	832	2,702	21,520	4,954
	Late abortion**	RHR3, 948 d	399 d in RHR3	1,162	2,825	19,643	6,831
Second pregnancy cow	Early abortion*	L1, 1,316 d	302 d in DP 1	782	2,351	21,520	4,954
	Late abortion**	DP1, 1,406 d	392 d in DP 1	1,102	2,194	19,643	6,831
Third pregnancy cow	Early abortion*	L2, 1767 d	300 d in DP 2	763	1,630	21,520	4,954
	Late abortion**	DP2, 1857 d	390 d in DP 2	1,087	1,414	19,643	6,831
Fourth pregnancy cow	Early abortion*	L3, 2,216 d	180 d in DP 3	605	1,035	22,730	3,744
	Late abortion**	DP3, 2,306 d	180 d in DP 3	613	782	22,730	3,744
Average	–	–	–	868	1,866		

NA, not applicable. In all scenarios, early (*) and late (**) abortions occur on days 135 and 225 of pregnancy. RHR: replacement heifer rearing, L: lactation, DP: dry period. *** Considering that the heifer or cow remains in the herd after aborting until the end of its productive life.

#### 3.2.2 Lactating and dry stages

In the “Lactation” and “Dry Cow” stages, differences were observed across abortion scenarios compared to the base scenario (Table 1). Regardless of the productive stage in which it occurred, abortion resulted in an earlier start of the new birth-(abortion-) conception interval and a delayed onset of the new lactation, thereby extending the “Dry Cow” stage. Table 1 presents the estimated individual economic losses for early and late abortions.

### 3.3 Direct economic losses from abortions caused by neosporosis at both individual and national levels in cows kept in the herd (no culling)

The estimated average value of individual losses from abortion due to neosporosis in cows within the herd was US$ 868 (Table 1). This linear average was calculated based on the differences in PV at the time of abortion for each scenario. Considering the individual economic loss from abortion associated with neosporosis for each scenario, as well as the annual number of abortions due to neosporosis and the distribution of abortion scenarios, we estimate that the direct economic losses (loss of profits) for the national primary dairy sector due to abortions from neosporosis in heifers and cows in the herd total US$ 9,997,519.

### 3.4 Direct economic losses from abortions due to neosporosis that result in the culling of cows at both individual and national levels

The estimated average value of individual losses due to abortion from neosporosis, considering the culling of the aborted dam, is US$ 1,866 (Table 1). This linear average was calculated based on the income from sales for slaughter minus the PV of a heifer or cow that did not abort, in accordance with the equivalent month for each abortion scenario described. The estimated economic losses (loss of profits) for the dairy primary sector due to neosporosis-related abortions, along with the culling of the aborting heifer or cow at the national level, amount to US$ 1,868,476.

### 3.5 Added direct economic losses for the primary sector at the national level due to abortions caused by neosporosis in cows that are kept in the herd and culled after abortion

The direct economic losses from abortions due to neosporosis in the primary dairy sector at the national level were calculated as the sum of losses across different abortion scenarios (pregnancy and early or late abortion) and sub-scenarios (92% maintenance and 8% culling). These losses amounted to US$ 11,865,995.

### 3.6 Differences in results across the different abortion scenarios for the economic response variables

The results of the economic response variables for different pregnancies indicate that losses were higher in first-pregnancy abortions (heifers). As the pregnancies progressed, the economic impact decreased. For the same pregnancy, losses were greater in late abortions compared to the early ones. Finally, among the eight simulated scenarios, the losses were highest for late abortions in the first pregnancy, as they showed the greatest difference in PV compared to the base scenario, along with the longest Payback Period and the lowest IRR. The difference in economic variables is explained by the impact of productive variables such as reduced lifetime production, the loss of offspring, and the devaluation of the cow over time, especially with regard to projected future milk production.

In addition to identifying differences in the response variables for the abortion scenarios compared to the base scenario and between abortion scenarios across different pregnancies, there were also differences among abortion scenarios for the same pregnancy, specifically between early and late abortions. Regarding individual losses, the difference in PV amounted to approximately US$ 320 of additional loss in the late scenarios for the first, second, and third pregnancies. An exception to this trend was noted in the fourth pregnancy’s abortion scenarios, where the difference was only US$ 8. This exception can be attributed to the fact that both scenarios share the same established end date for the productive life (day 2,544), meaning that the losses cannot be accumulated or expressed in the subsequent productive stage as they can in other scenarios.

In each simulated abortion scenario, there were economic and productivity losses that varied in magnitude. However, when evaluating the possibility of retaining or immediately culling the aborted cow, certain outcomes indicate reduced losses. There are situations where, from an economic perspective, it is advisable to keep the dam in the herd, while in other cases, culling is recommended (Table 1). This information can assist in making production decisions, although the economic results would only be valid under the assumptions used in this study; therefore, it should be approached with caution. The scenarios in which it would be advisable to retain the aborted dam in the herd include “heifer early abortion,” “heifer late abortion,” “second pregnancy cow early abortion,” and “third pregnancy cow early abortion.” In all other scenarios, it would be appropriate to cull the aborted cow. Generally, based on these assumptions, it can be concluded that the older the cow, the more beneficial it is to cull her rather than keep her after an abortion. For the scenarios of “second pregnancy cow late abortion” and “third pregnancy cow early abortion,” the differences between retaining and culling are much smaller compared to the others, warranting a more detailed evaluation.

In the case of abortion in heifers, it should be emphasized that it would be advisable to keep them in the herd under the assumption that these animals will experience only one abortion during their lifetime. However, it should be noted that there is a risk of these animals suffering more than one abortion due to neosporosis (25). In such cases, further estimations should be conducted to better assess whether keeping these animals is more economically viable than culling them. Additionally, even if seropositive cows do not abort, they are more likely to give birth to congenitally infected calves (vertical transmission), contributing to the transgenerational spread of the disease within the herd. If these congenitally infected animals are female, they are also at a higher risk of abortion due to neosporosis. These transgenerational epidemiological effects were not incorporated into the model used in this study and warrant further exploration before outlining recommendations regarding the merits of maintaining or culling cows that abort as a result of neosporosis.

The neosporosis abortion scenarios simulated here represent various ages, parities, and lactations. Furthermore, distinctions are made between early and late abortions within the same pregnancy, considering the gestational window during which the highest frequency of abortion is reported for neosporosis. Regarding the relationship between the seroprevalence of neosporosis and the age of the heifers and cows, a direct relationship has been described (8, 32, 33); however, some studies have indicated an inverse relationship (34). Regarding abortion and age in seropositive dams, contradictory results have been observed, showing trends toward both direct (29) and inverse relationships (28). It has been suggested that the risk of abortion is generally higher in older seropositive cows, although some herds with endemic neosporosis have identified a greater risk in younger animals (25, 33, 35, 43). This phenomenon could be explained by the predominant transmission route and the management decisions made by farmers regarding seropositive dams (25, 28, 35). The distribution of abortion scenarios simulated in the Uruguayan national herd is based on the endemicity of the disease. The seroprevalence of neosporosis in Uruguay has remained at consistent levels for decades (10, 36, 37), and the reliance on locally bred calves as replacement heifers supports a successful vertical transmission cycle and the maintenance of the endemic situation. However, the lack of local data concerning the characterization of cows with abortions specifically attributable to neosporosis has led us to adopt a conservative position and assume a uniform distribution of total abortions across the scenarios in heifers, as well as first, second, and third lactation cows.

### 3.7 Productive losses due to neosporosis at the individual and national levels

Productive losses include lost calves and milk. Regarding lifetime milk production during the abortion scenarios of the first pregnancy, the figures were 21,520 L and 19,663 L for early and late abortions, respectively, indicating differences of −4,953 L and −6,810 L compared to the base scenario (with a lifetime production of 26,474 L) (Table 1). The difference between the early and late abortion scenarios was 1,856 L, favoring early abortion. In the second and third-pregnancy abortion scenarios, production remained consistent with the first-pregnancy scenarios for both early and late abortions, resulting in identical differences between the scenarios. For the fourth pregnancy abortion scenarios, production was 22,730 L for both early and late abortions, which led to a difference of −3,744 L compared to the base scenario (Table 1).

Regarding the calves, considering the number of annual abortions due to neosporosis, a total loss of 12,510 calves occurred nationwide in 2018. Of the estimated number of heifers and cows that were aborted because of neosporosis, we assumed that 11,509 (92%) were kept in the herd until the end of their productive lives. Considering the distribution of abortion scenarios and the variations in lifetime milk production for each scenario compared to the base scenario, the total losses from abortions in 2018 amounted to 61,637,560 L of milk; these losses are expected to accumulate over the 7 years following 2018 (until 2025).

The effects of abortion on productivity, reproduction, and economic factors have been examined using various methodologies. Studies have employed diverse approaches to consider abortion either as a syndrome (irrespective of the cause) or as a clinical sign of a specific disease. When viewed as a syndrome, productive costs increase under the following circumstances: (1) early culling of the cow, (2) an increase in unproductive days, leading to higher maintenance expenses, (3) delays in subsequent lactations or the loss of an entire lactation, (4) decreased milk production during the lactation in which abortion occurs or during the new lactation that prematurely begins after abortion (20, 38–41).

Regarding early culling and abortion, a direct relationship has been established. Keshavarzi et al. (41) estimated an increase in the risk of culling due to general health problems or reproductive issues by 1.89 and 2.41 times (*p* < 0.01) compared to cows that did not experience abortion. Regarding culling and seropositivity for neosporosis, it has been estimated that seropositive female cows have a 1.6- to 1.7-fold greater likelihood of being culled compared to seronegative ones during the 3 years following the abortion (*p* = 0.01, 0.04) (30). Based on these findings, Chi et al. (21), Häsler et al. (15), and Liu et al. (17) calculated the economic impact of early culling due to neosporosis at the herd level as an additional culling rate of 2% annually for seropositive female cows. Although this method has been utilized over the years, it does not consider abortion as a variable that directly affects culling. In our study, we included the economic effect of early culling due to abortion in *N. caninum* seropositive dams based on the study by Thurmond and Hietala (30). This analysis determined that, of the total number of seropositive heifers and cows that experienced abortions, 8% were culled early. Additionally, seropositive dams that aborted had a threefold greater risk of culling. In our study, this value accounted for seropositive dams that were culled due to abortions; for the remaining 92%, we assumed that they remained in the herd until the end of their productive life, with the limitation that this does not necessarily reflect the Uruguayan reality.

The economic loss from culling has been estimated as the replacement cost minus the slaughter sale value (13, 15, 17, 21). However, these estimates are likely understated, as the loss of the female has not been considered in PV terms regarding her stage and future production, which is further diminished by the sale value from culling and slaughter. Our estimate of the economic loss from abortions due to neosporosis, including the culling of the aborted dam, is higher than those from earlier studies, possibly due to these factors. Even when estimates are made at the national herd level, culling will not mitigate total losses nationwide, as there is no true replacement for the culled animal; rather, at best, there is a replacement from one dairy farm to another. The indirect effects on the integrity and sustainability of Uruguayan dairy farming were not estimated in our study. In our analysis, the estimate of the economic loss resulting from an abortion that prompted culling considered the sale for slaughter, suggesting a reduction in the cow stock at the national level.

Regarding the economic losses associated with cows that abort due to neosporosis, studies are limited. Reichel et al. (33) estimated the individual loss at NZ$ 900 (US$ 560) in New Zealand. In Argentina, the estimated loss due to abortion was US$ 1,415 (13). Both results fall outside the expected range but are not significantly different from our study’s findings (US$ 605–1,160) in sub-scenarios where the aborting dam remained in the herd. The relative similarities in these outcomes may be attributed to the comparable grazing-based dairy systems of New Zealand, Argentina, and Uruguay. In a study by Liu et al. (17) in China, individual losses due to abortion from neosporosis ranged from US$ 524 to US$ 2,337. In our analysis, values exceeding US$ 2,000 were achieved only when simulating specific scenarios of abortion coupled with culling (US$ 782–2,825).

Numerous studies have highlighted the annual economic losses caused by neosporosis at the herd level. In Switzerland, Häsler et al. (15) estimated these losses at €9.7 million for a herd of 1,024,285 animals at risk of abortion. In the Netherlands, annual losses were estimated to be €19 million for a herd of 1,500,000 susceptible animals (15). In Argentina’s humid Pampa region, the annual losses due to abortions from neosporosis in dairy cattle were estimated at US$ 33,097,221, accounting for 23,382 abortions (13). A more recent study in the same region found that annual losses from neosporosis in dairy cattle totaled US$ 10,537,465, factoring in 7,447 abortions (42). In a study examining a herd of only 50 animals in Canada, Chi et al. (21) estimated the annual economic impact of neosporosis at US$ 2,304.

Reichel et al. (14) estimated the annual economic losses caused by neosporosis in dairy and beef cattle within the primary sector at the national herd level across 10 countries. This estimation considered the female cows at risk of abortion due to neosporosis based on the bibliographic information available in each country. The corresponding annual losses in millions of US$ for dairy and beef cattle were 38.5 and 48.9 in Argentina, 26.6 and 74.1 in Australia, 51.3 and 101 in Brazil, 17.1 and 14.3 in Canada, 68.5 and 94.8 in Mexico, 12.1 in The Netherlands (for dairy cattle only), 35.7 and 1.1 in New Zealand, 19.8 and 9.8 in Spain, 27.0 in the United Kingdom (for dairy cattle only), and 546.3 and 111.4 in the US. In total, the losses for the dairy and primary beef sectors were US$ 842 million and US$ 455.4 million, respectively. Overall, the annual losses amounted to US$ 1,298 million. This study is the first to estimate the economic losses from abortion and neosporosis in Uruguay.

The economic model we used considered many key variables and input data. However, we acknowledge that both the model and the simulations have limitations. We have reasons to believe that the economic loss results in this study are underestimated and provide a baseline magnitude for losses due to neosporosis at the national level.

First, the model considers only dairy (but not beef) cattle breeds. It even overlooks the economic losses that dairy farmers face due to reduced beef production; specifically, losses related to breeding, fattening and the sale of male calves from dairy breeds that are lost due to neosporosis. Furthermore, due to its deterministic approach, the results lack probabilistic support because they do not account for uncertainty by constructing confidence intervals, even though the calculations that determine the results are well-founded. The individual model assumes that a single abortion occurs over the productive life of a cow and that the post-abortion birth-conception interval will reflect that of the average cow in Uruguay, which does not capture the variability of reproductive decisions made by farmers when faced with an abortion event. Regarding the epidemiological dynamics of neosporosis and its economic effects, we have not considered the impact of vertical transmission on the transgenerational spread of the disease, as the model assumes that each offspring will be sold. Additionally, we have not factored in the decrease in breeding value and the consequent loss of genetic merit of the cow that aborted and was removed from the herd or the unborn female calves that perish in the fetal stage, the effects of stunted growth in the national dairy cattle herd, or any other indirect economic losses. For several of our input data, we relied on expert opinion due to the limited availability of scientific articles or statistics with local data regarding the characterization of cows that abort, their reproductive efficiency, and milk production post-abortion. Finally, our estimates have focused on the direct economic losses incurred by the primary dairy sector, implying that we have not quantified the economic losses from the lost profits of the liters of milk not received or processed by the dairy industry (secondary sector) nor have we estimated the losses of products or services provided by the tertiary sector.

## 4 Conclusion

In this study, we estimated the annual economic losses in the primary dairy sector due to abortions caused by neosporosis within Uruguay’s national dairy herd. Despite the challenges and limitations of the model, the losses were significant. The economic and productive loss data could be used to assess the financial feasibility of implementing control programs for this disease in Uruguay.

## Data Availability

The raw data supporting the conclusions of this article will be made available by the authors, without undue reservation.
