# Supplementary material for: Estimation of direct economic and productive losses due to abortions caused by Neospora caninum in the primary dairy sector of Uruguay
Source: Front Vet Sci. 2025 Mar 26;12:1502742. doi: 10.3389/fvets.2025.1502742 (PMC11979231; doi:10.3389/fvets.2025.1502742)
Supplement: Supplementary file 1 [file Supplementary_file_1.docx]

**Supplementary Material I**

Definition of variables

The Total Accumulated Balance includes income, costs, investments, and amounts recovered at the end of the lifespan. The Accumulated Balance at the end of each stage represents the same but until that moment. The Accumulated Balance allows determining when the costs incurred are covered (Payback Period or Recovery of Expenses). The Payback Period occurs at different times depending on the simulated scenario. This was calculated using the following formula:

Month_n_ + AB_n_ / (AB_n_ – AB_n-1_)

“Month_n_” is the month in which the Accumulated Balance becomes positive for the first time, “AB_n_” is the Accumulated Balance of month n and “ABn-1”is the Accumulated Balance of the previous month.

The Present Value (PV) is the sum of the animal's future income and expenses updated at a given moment (Huirne et al., 1997; Rushton, 2009). It indicates how much each animal is worth during each month of its productive life and allows estimating how much would be lost if the cow died in each of the stages of the productive life.

The PV refers to the following expression:

$$VA= \sum_{lifespan}^{n=1} \frac{{Sf}_{n}}{{(1+i)}^{n}}$$

Being “n” each of the remaining months of the animal's lifespan, “Sfn” each of the balances (income - expenses) of month “n”, and “i” the interest rate assigned as the discount rate for each period. We assumed that the annual discount rate corresponded to an opportunity cost of capital (for the purposes of valuing the time effect) estimated at 5% (0.4167% monthly, i = 0.004167). The PV of a future cash flow represents how much money would have to be invested in the present moment at the interest rate “i” to obtain that cash flow over future time.

The difference in PV between the base scenario and each abortion scenario (see below) represents the economic impact of abortion in a broad sense. The economic loss due to abortion was determined by calculating the PV of the future production that is no longer obtained, minus the direct costs that are avoided by this event (lost profits). We applied a marginal approach to determine the economic losses due to abortion by estimating the difference between the PV of a female in the case of no abortion (base scenario) and the PV in an abortion scenario (Gädicke et al., 2010).

That is, the economic loss due to abortion (LA) was calculated as: LA = PV cow with abortion - PV cow without abortion.

The internal rate of return (IRR) is a measure of the profitability of an investment project that expresses the interest rate at which the present value of future benefits equals the present value of costs. This is, the interest rate that would have to be applied to the cash flow from the project so that its net present value (NPV) is zero (Huirne et al., 1997). Because it is expressed as a proportion, it is useful to reaffirm the results obtained from the measurements and comparisons of the current values of each scenario and confirm the lower profitability at a global level due to abortion and its consequences. In our case, it was calculated as the interest rate that leaves the PV of the total future cash flow at zero (at the birth of the animal) minus an initial investment corresponding to the value proposed for a newborn female calf (US$ 100). In this study, the PV determines the value of the cow as a function of time.

Expenditures include both expenses and investments. Expenses reflect expenses for food, health, labor, and reproduction. The investment corresponds to the amount that must be invested in facilities and equipment if necessary for each production stage. For these, the estimated useful life of each asset is taken into account, and it is considered that the expenses take place at the beginning of the stage in which they are necessary. Furthermore, it is assumed that those required for Replacement Heifer Rearing 1 are also used in Replacement Heifer Rearing 2 and 3. These investments, for all scenarios, were US$ 1.42, 5.38 and 876.92 for the Calf Rearing, Replacement Heifer Rearing 1 and Lactation 1 stages, respectively (see Supplementary Material II).

The income includes dairy production, the sale value of the calf (US$ 61 per calf, which is the average between the price of the male and the female), the sale of the cow by discarding and the partial recovery of the investments when they stopped being used by the animals in the different stages. The recovery of investments corresponds to the residual value of the facilities at the end of the useful life of the animal.

The economic response variables were PV, IRR and Payback Period, while the productive response variables were lifetime milk production and the number of offspring.

**Supplementary Material II**

Supplementary Table 1. Generic input data.

| **Input Data** | **Value** | **Source** |
| --- | --- | --- |
| Labor costs | 14,955 US$/año | 1 |
| Breeding stage food cost | 0.4235 US$/kg DM | 2 |
| Rearing 1 stage food cost | 0.4 US$/kg DM | 2 |
| Rearing 2 and 3, Lactation and Dry Cow stage food costs | 0.3294 US$/kg DM | 2 |
| Hay cost in all productive stages | 0.0339 US$/kg DM | 1, 2, 3 |
| Permanent grasslands costs | 0.041 US$/kg DM | 1, 2, 3, 4 |
| Raw milk price | 0.30 US$/L | 1 |
| Female calf sale price | 100 US$ | 5 |
| Male calf sale price | 22 US$ | 5 |
| Culled cow sale price | 600 US$ | 5 |

DM: dry matter.

Sources: 1- INALE 2014, 2-PROLESA, 3-Expert opinions, 4- Caffarena 2021, 5- Auction House.

Supplementary Table 2. Input data by productive stage from Calf Rearing to Replacement Heifer Rearing 3.

| **Input data by productive stage US$/head** | **Calf Rearing** | **Replacement Heifer Rearing 1** | **Replacement Heifer Rearing 2** | **Replacement Heifer Rearing 3** | **Source** |
| --- | --- | --- | --- | --- | --- |
| Labor costs | 43.0 | 5.05 | 25.96 | 13.0 | 1 |
| Heating liquid food cost | 1.82 |  |  |  | 5 |
| Grazing feeding cost |  |  | 0.00 | 0.00 | 1 |
| Grain feeding cost | 18.55 | 61.42 | 57.41 | 142.56 | 1 |
| Hay feeding cost | 0.12 | 3.67 | 21.26 | 28.64 | 1, 2, 3 |
| Permanent pasture feeding cost |  | 1.60 | 59.70 | 20.80 | 1, 2, 3 |
| Corn silage feeding cost |  |  |  | 34.15 | 1, 2 |
| Navel asepsis cost | 0.2 |  |  |  | 2, 3 |
| Parasite treatment cost |  | 0.321 | 0.64 |  | 2, 3 |
| Neonatal diarrhea treatment cost | 18.78 |  |  |  | 2, 3 |
| Veterinary services cost | 5.14 | 0.68 | 1.37 | 0.68 | 4, 6 |
| Anthrax vaccine cost |  | 0.420 |  |  | 4, 6 |
| Foot and mouth disease vaccine cost |  | 0.00 | 0.00 |  | 4, 6 |
| Clostridial diseases vaccine cost |  | 1.01 | 2.02 |  | 4, 6 |
| Brucellosis vaccine and Tuberculosis diagnosis yearly cost |  |  | 6.93 | 6.93 | 4, 6 |
| Reproductive vaccine cost |  |  | 3.00 |  | 4, 6 |
| Semen cost |  |  | 15.00 |  | 2 |
| Insemination cost |  |  | 2.62 |  | 2, 6 |
| Pregnancy diagnosis cost |  |  | 1.94 |  | 4, 6 |
| Water tank investment |  | 5.38 |  |  | 3 |
| Stakes investment | 0.25 |  |  |  | 3 |
| Facilities investment | 1.00 |  |  |  | 3 |
| Hot water tank investment | 0.17 |  |  |  | 3 |

Sources: 1- INALE 2014, 2-PROLESA, 3-Expert opinions, 4- Caffarena 2021, 5- Auction House, 6- Veterinary Medical Association of Uruguay.

Supplementary Table 3. Input data by productive stage from Lactation 1 to Lactation 4.

| **Input data by productive stage US$/head** | **Lact. 1** | **Dry Period 1** | **Lact. 2** | **Dry Period 2** | **Lact. 3** | **Dry Period 3** | **Lact. 4** | **Source** |
| --- | --- | --- | --- | --- | --- | --- | --- | --- |
| Labor costs | 250.92 | 40.02 | 247.11 | 39.38 | 245.84 | 39.38 | 114.34 | 1 |
| Grazing feeding cost |  | 0.00 |  | 0.00 |  | 0.00 |  | 1 |
| Grain feeding cost | 549.77 | 16.80 | 641.76 | 36.96 | 638.60 | 35.28 | 301.21 | 1 |
| Hay feeding cost | 12.12 | 3.50 | 13.31 | 4.81 | 13.53 | 4.70 | 6.34 | 1, 2, 3 |
| Permanent pasture feeding cost | 105.43 | 2.85 | 130.56 | 2.81 | 125.53 | 2.81 | 67.46 | 1, 2, 3 |
| Corn silage feeding cost | 86.67 | 5.49 | 95.12 | 12.08 | 96.74 | 11.53 | 45.33 | 1, 2 |
| Anthrax vaccine cost | 0.42 |  | 0.42 |  | 0.42 |  | 0.42 | 4, 6 |
| Foot and mouth disease vaccine cost | 0.00 | 0.00 | 0.00 | 0.00 | 0.00 | 0.00 | 0.00 | 4, 6 |
| Brucellosis vaccine and Tuberculosis diagnosis yearly cost | 6.93 | 6.93 | 6.93 | 6.93 | 6.93 | 6.93 | 6.93 | 4, 6 |
| Reproductive vaccine cost | 1.50 |  | 1.50 |  | 1.50 |  | 1.50 | 4, 6 |
| Semen cost | 15.00 |  | 15.00 |  | 15.00 |  | 15.00 | 2 |
| Insemination cost | 2.62 |  | 2.62 |  | 2.62 |  | 2.62 | 2, 6 |
| Other costs (including milking) | 48.19 |  | 54.41 |  | 56.51 |  | 26.21 | 5, 3 |
| Water tank investment | 107.69 |  |  |  |  |  |  | 3 |
| Feeders investment | 153.85 |  |  |  |  |  |  | 3 |
| Refrigeration equipment investment | 153.85 |  |  |  |  |  |  | 3 |
| Milking equipment investment | 130.77 |  |  |  |  |  |  | 3 |
| Milking facilities investment | 330.77 |  |  |  |  |  |  | 3 |
| Calf sale income | 61.00 |  | 61.00 |  | 61.00 |  | 61.00 | 5, 3 |
| Dairy production income | 1989.03 |  | 2245.39 |  | 2332.24 |  | 1081.64 | 1, 3 |

Sources: 1- INALE 2014, 2-PROLESA, 3-Expert opinions, 4- Caffarena 2021, 5- Auction House, 6- Veterinary Medical Association of Uruguay.

**Equations for calculating input data**

1. Calculation of the Residual Value (RV) of fixed assets:

$$RV=\left( VN-RI \right) . \frac{future duration}{lifespan}+RI$$

VN is the value of the new asset and RI is the residual income.

2. Calculation of labor costs in the Calf Rearing stage.

$$Labor cost per head for the Calf Rearing stage = \frac{Annual salary}{(\frac{Cohort}{year}\times\frac{Animals}{Cohort})}$$

3. Calculation of the cost of treatment of neonatal diarrhea syndrome.

$$Curative healthcare = cost of diarrhea treatment \left( \frac{US\$}{treatment} \right)$$

$$x Diarrhea Morbility\left( \frac{affected}{population at risk} \right)$$

4. Equations for calculating the cost of labor per head from Replacement Heifer Rearing 1 stage to Lactation 4 stage.

$$Labor cost per head for the stage=\frac{\frac{hours}{head}}{stage} \times salary per hour of work$$

$$\frac{\frac{hours}{head}}{stage} =\frac{{Working hours}/{Year}}{heads/person}\times\frac{Stage duration}{365}$$

5. Calculation of cost of insemination for stages Replacement Heifer Rearing 2, Lactation 1, 2, 3 and 4.

$$Insemination cost:\left( Semen price+insemination cost/dose \right)\times N^{\circ}{dose}/{head}+{insemination cost}/{Pregnancy}\times Pregnancy Rate$$

**Input data for temporary variables in abortion scenarios**

Supplementary Table 4. Duration of the different productive stages of each scenario in days.

| **Scenario** | Base (No. abortion) | Heifer early abortion | Heifer late abortion | Cow second pregnancy early abortion | Cow second pregnancy late abortion | Cow third pregnancy early abortion | Cow third pregnancy late abortion | Cow fourth pregnancy early abortion | Cow fourth pregnancy late abortion |
| --- | --- | --- | --- | --- | --- | --- | --- | --- | --- |
| **Productive**  **Stage** |  |  |  |  |  |  |  |  |  |
| **Calf Rearing** | 73 | 73 | 73 | 73 | 73 | 73 | 73 | 73 | 73 |
| **Replacement Heifer Rearing 1** | 107 | 107 | 107 | 107 | 107 | 107 | 107 | 107 | 107 |
| **Replacement Heifer Rearing 2** | 550 | 550 | 550 | 550 | 550 | 550 | 550 | 550 | 550 |
| **Replacement Heifer Rearing 3** | 276 | 585 | 675 | 276 | 276 | 276 | 276 | 276 | 276 |
| **Lactation 1** | 395 | 395 | 395 | 395 | 395 | 395 | 395 | 395 | 395 |
| **Dry Period 1** | 63 | 56 | 56 | 365 | 455 | 63 | 63 | 63 | 63 |
| **Lactation 2** | 389 | 389 | 389 | 389 | 389 | 389 | 389 | 389 | 389 |
| **Dry Period 2** | 62 | 60 | 60 | 60 | 60 | 362 | 452 | 62 | 62 |
| **Lactation 3** | 387 | 329 | 239 | 329 | 239 | 329 | 239 | 387 | 387 |
| **Dry Period 3** | 62 | 0 | 0 | 0 | 0 | 0 | 0 | 242 | 242 |
| **Lactation 4** | 180 | 0 | 0 | 0 | 0 | 0 | 0 | 0 | 0 |
